# Dermatomyositis-Induced Rhabdomyolysis With Features of Necrotizing Myopathy and Acute Inflammatory Demyelinating Polyneuropathy in an Epstein-Barr Virus Infected Patient

**DOI:** 10.7759/cureus.12077

**Published:** 2020-12-14

**Authors:** Ammar Haikal, Swati Govil, Arsany Anis, Jenna Guma

**Affiliations:** 1 Internal Medicine, Hackensack Meridian Medical Center, Hackensack, USA; 2 Internal Medicine, Hackensack Meridian Health Palisades Medical Center, North Bergen, USA; 3 Internal Medicine, Saint Michael's Medical Center, Newark, USA; 4 Internal Medicine, Rowan University School of Osteopathic Medicine, Stratford, USA

**Keywords:** inflammatory myopathy, dermatomyositis, necrotizing myopathy, acute inflammatory demyelinating polyneuropathy, epstein-barr virus, elevated creatine phosphokinase (cpk)

## Abstract

Dermatomyositis (DM) is an autoimmune inflammatory myopathy characterized by features of a typical rash, proximal muscle weakness, and evidence of muscle inflammation. Acute inflammatory demyelinating polyneuropathy (AIDP) is an autoimmune peripheral nerve disease characterized by myelin damage and progressive areflexic weakness and sensory changes. AIDP can be precipitated by viral infections such as Epstein-Barr virus (EBV). We present a case of DM with rhabdomyolysis and necrotizing features, along with AIDP in the setting of EBV viremia. DM and AIDP rarely coincide together. The patient was treated with a combination therapy of methylprednisolone, azathioprine, and intravenous immunoglobulins (IVIGs), which led to significant improvement in his symptoms.

## Introduction

Dermatomyositis (DM) is an autoimmune inflammatory myopathy (IM) characterized by features of a typical rash, proximal muscle weakness, and evidence of muscle inflammation. Acute inflammatory demyelinating polyneuropathy (AIDP) is an autoimmune peripheral nerve disease characterized by myelin damage and progressive areflexic weakness and sensory changes. AIDP can be precipitated by viral infections such as Epstein-Barr virus (EBV). We present a case of DM with rhabdomyolysis and necrotizing features, along with AIDP in the setting of EBV viremia. DM and AIDP rarely coincide together. The patient was treated with a combination therapy of methylprednisolone, azathioprine, and intravenous immunoglobulins (IVIGs), which led to significant improvement in his symptoms.

A consent was obtained to show the patient's face.

## Case presentation

**Figure 1:**
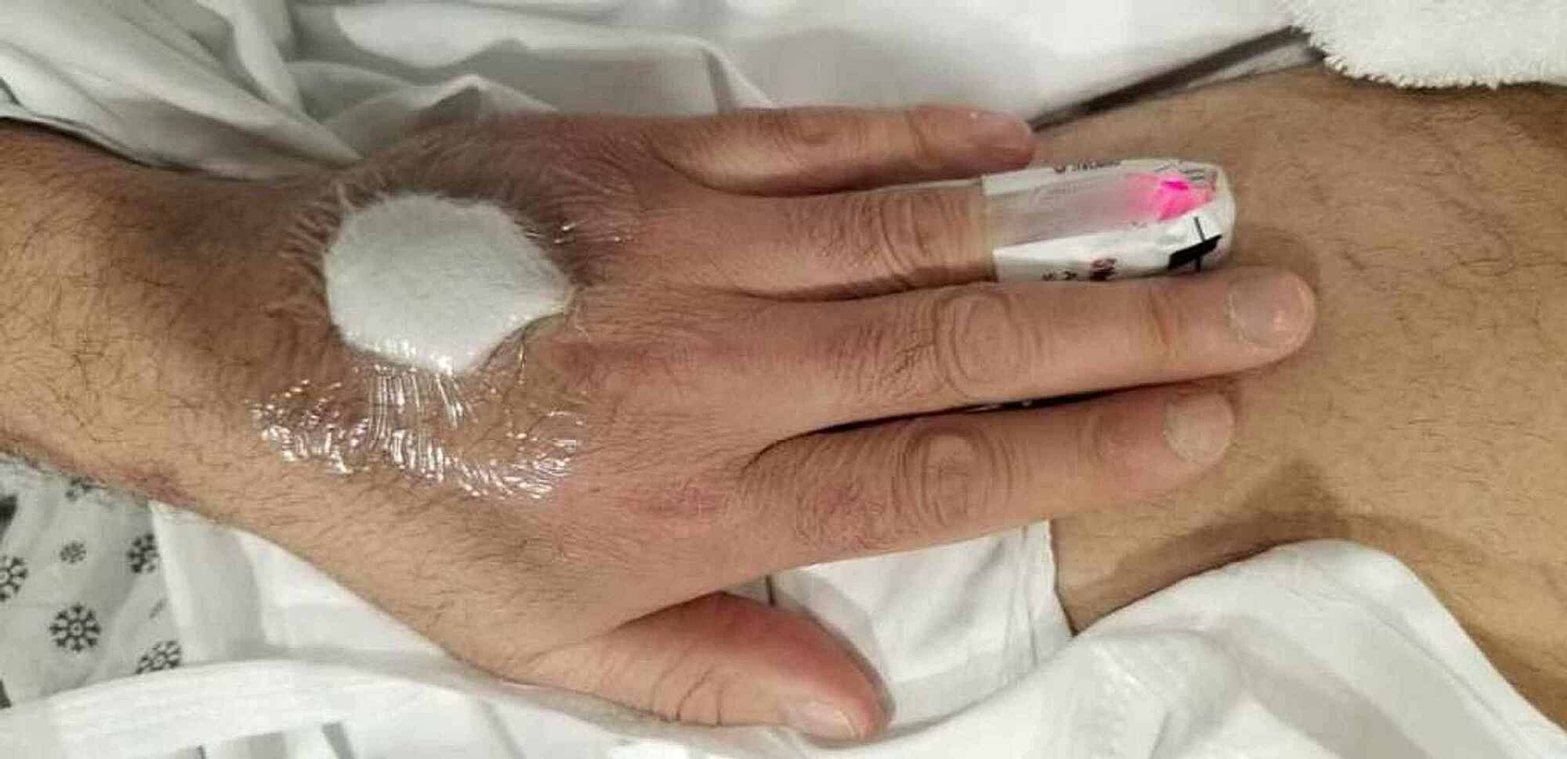
Gottron's papules

**Figure 2:**
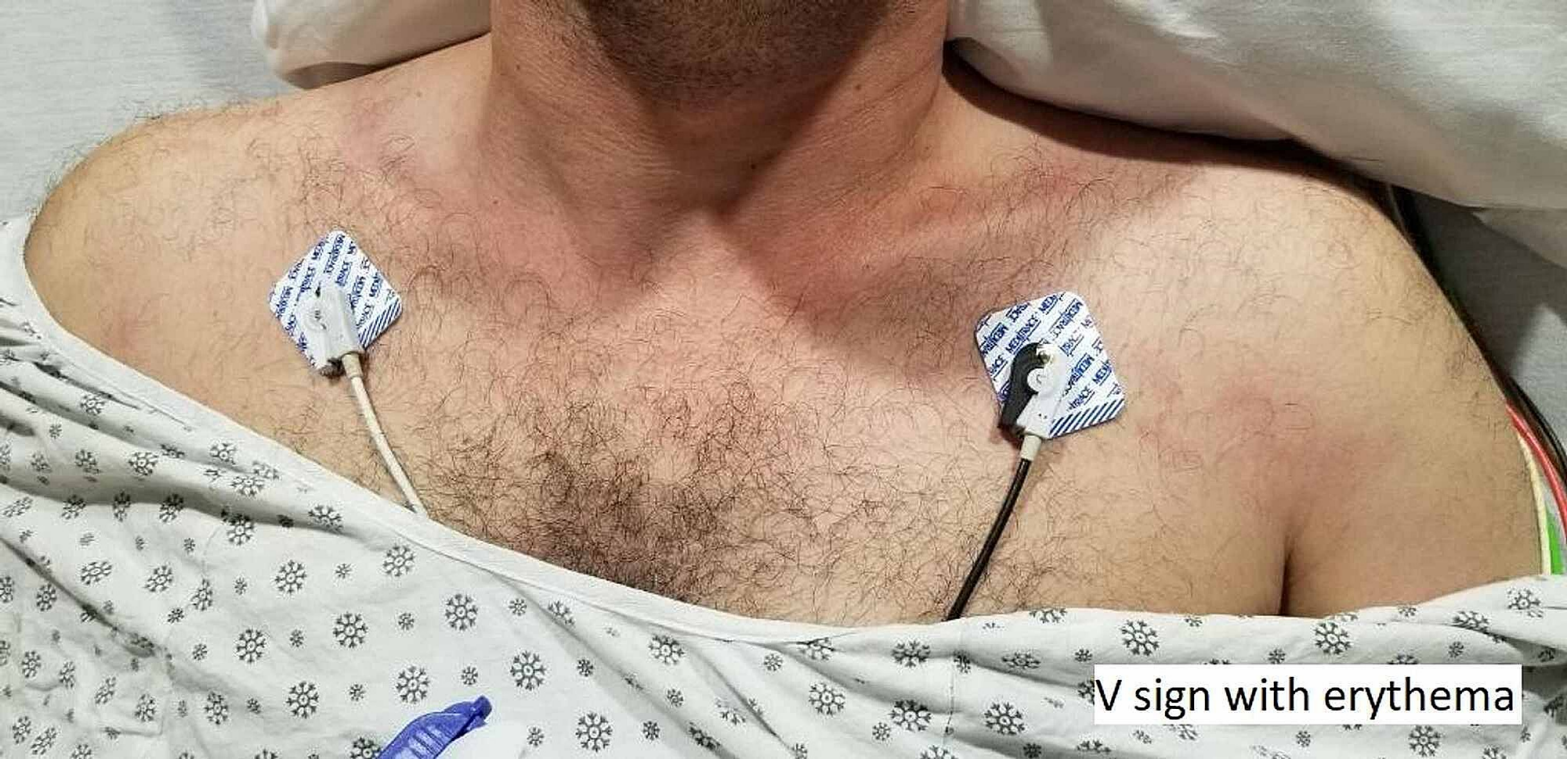
Shawl sign

**Figure 3:**
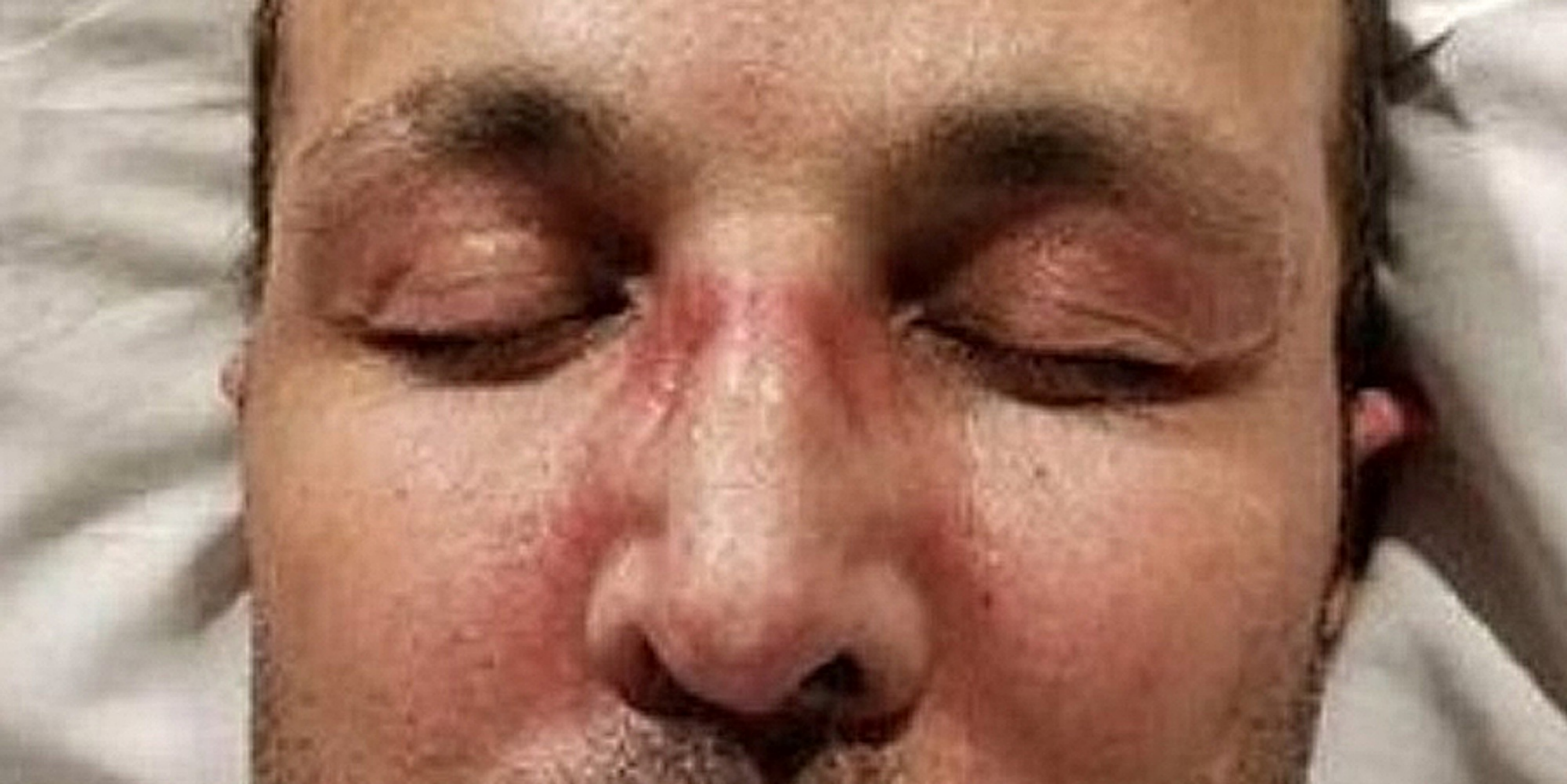
Heliotrope rash

A 49-year-old Middle Eastern man with heterozygous factor V Leyden deficiency and previous deep venous thrombosis (DVT) on Apixaban developed progressively worsening symmetric proximal upper and lower extremity muscle weakness and dysphagia. While he was scheduled for a routine cardiac stress test, he had elevated creatine phosphokinase (CPK) at 19,000 units per liter (U/L) (normal range: 49-439 U/L), aspartate transaminase (AST) of 500 iU/L (normal range: 0-40 U/L), and alanine transaminase (ALT) 171 iU/L (normal: 0-44 U/L) for which he was admitted. Erythrocyte sedimentation rate (ESR) and C-reactive protein (CRP) were 38 mm/hour and 16.5 mg/L, respectively (normal values: 0-15 mm/hour and 0-10 mg/L, respectively). An MRI of the lower extremity showed symmetric bilateral muscle edema throughout the pelvis, suspicious for myositis per radiology report. The patient was treated supportively with intravenous (IV) hydration and IV methylprednisolone 40 mg twice a day for three days by an internal medicine team. He underwent muscle biopsy from the left thigh, which was pending on discharge. A barium swallow evaluation showed continuously regurgitated residual material. A CT of the chest, abdomen, and pelvis showed no evidence of malignancy. Prostate-specific antigen (PSA) three months prior to presentation was normal. Myositis panel was positive for Mi-2 alpha and beta antibodies (56 and 39, respectively; normal < 11), and negative for anti-signal recognition particle (SRP), transcriptional intermediary factor 1 (TIF) antibodies, and rest of the antibodies on the myositis panel (LabCorp myositis panel II). He improved clinically, and CPK trended down to 7,000 iU/L on discharge. The patient was discharged on 100 mg methylprednisolone. He was referred to our rheumatology clinic, and azathioprine was added. The muscle biopsy results showed large necrosis and phagocytosis. There was some localized necrotic fiber in other areas and no evidence of hemorrhage or vessel occlusion. Cluster of differentiation 45 (CD45) stain showed lymphocytes in the area of necrosis mainly. Major histocompatibility complex (MHC) was also upregulated in a patchy way. Nicotinamide adenine dinucleotide hydrogen (NADH) stain showed no significant diagnostic abnormalities. Periodic acid-Schiff (PAS) stain showed no glycogen accumulation in the preserved area of muscle and no lipid accumulation. No congophilic material was seen. A follow-up CPK at the time of his initial visit was 26,000 iU/L despite being on 100 mg of methylprednisolone orally daily; therefore, the patient was hospitalized again. In the hospital, the neurology and rheumatology services were consulted. Physical examination was notable for areflexia and weakness in the upper extremities (neck flexion 3/5, neck extension 4/5, deltoid abduction 1/5, biceps flexion 4/5, hip flexion 2/5, hip extension 4/5, and normal upper and lower extremity strength distally). During this admission, he developed a rash suggestive of Gottron’s papules, shawl rash, and heliotrope rash, as illustrated in Figures 1-3. He was subsequently treated with one gram of methylprednisolone for three days and IVIGs 2 mg/kg for five days given dysphagia and rhabdomyolysis. His CPK peaked at 36,000 iU/L, with an ALT and AST of 413 U/L and 1,112 U/,L respectively. An electromyography (EMG) and nerve conduction study showed evidence of both sensorimotor axonal degeneration and demyelinating polyneuropathy. The EMG also showed significant spontaneous fibrillations, decreased motor unit action potential duration, and decreased amplitude in the deltoid and iliopsoas muscles, which were classic features consistent with AIDP. Skin biopsy of the left upper chest and the right dorsal hand showed interface dermatitis and features consistent with Gottron's papules, respectively. As part of the work-up for rhabdomyolysis, EBV PCR (polymerase chain reaction) was elevated at 89 IU/mL. Other extensive infectious work-up including cytomegalovirus was negative. After discharge, he followed with rheumatology and a neuromuscular specialist. Colonoscopy was recommended. Two months after discharge, his CPK normalized. Dysphagia and muscle strength also significantly improved while on methylprednisolone taper and azathioprine 150 mg (1.8 mg/kg). However, azathioprine was discontinued later due to leukopenia (neutrophil count below 500), and the patient was maintained only on IVIG and 4 mg of methylprednisolone for a total of four months. Subsequently, he had a relapse of unclear etiology, and CPK was 14,000 iU/L on routine check. The patient did not have significant weakness but complained of myalgia in the thighs. He was admitted for hydration, glucocorticoid was tapered, and mycophenolate mofetil was started. CPK peaked at 53,000 iU/L. He continues to receive monthly IVIG, mycophenolate mofetil 1,000 mg twice daily, and methylprednisolone 4 mg daily. CPK continues to trend down, which was 438 iU/L at the time of writing this case (11 weeks following discharge).

## Discussion

IM includes DM, polymyositis, antisynthetase syndrome, immune-mediated necrotizing myopathy (IMNM), and inclusion body myositis. The incidence of this group of diseases is one per 100,000 populations [1]. In DM, patients usually present with symmetrical muscle weakness of proximal muscle groups such as deltoid muscles, hip flexors, or neck flexors muscles. It tends to be mild to moderate. On the other hand, IMND usually presents with severe proximal weakness, lower extremity weakness, and severe fatigue. It very rarely presents with dysphagia and respiratory muscle weakness [2]. IMNM comprises only 16% of IM [2].

Diagnosis of DM is made based on a combination of clinical pictures, laboratory findings, imaging, and muscle and/or skin biopsy. In IM, creatine kinase (CK) level is the most sensitive measure but does not correlate with the severity of the symptoms. In DM, 70-80% will have up to 50-fold levels, whereas 20% of DM patients will have normal CK levels. CK levels in IMNM can be extremely high and reach 100-fold (around 10,000 iU/L) [3]. Various case reports on IMNM describe CK levels rising to around 28,000 iU/L. In one of the largest case studies, the mean peak CK reported for each of a variety of different causes ranged from approximately 10,000 to 25,000 iU/L [4]. To our knowledge, our patient’s CK levels were one of the highest recorded, at 36,000 then 53,000 iU/L on relapse.

Anti-Mi-2 antibodies are strongly associated with DM (frequency up to 31%). Autoantibodies targeting the Mi-2 nuclear antigen represent one of the serologic hallmarks of idiopathic IMs, with a diagnostic sensitivity and specificity of approximately 4-18% and 98-100%, respectively [3]. DM patients positive for this antibody are reported to have typical skin lesions (Gottron’s papules, heliotrope rash, shawl sign, and V-sign) and myositis, as seen in our patient. Patients with anti-Mi-2 antibodies typically respond well to steroid therapy and have a good prognosis. Those cases are rarely associated with internal malignancy and interstitial lung disease [3].

EMG studies of DM patients show increased spontaneous and insertional activity with fibrillation potentials, positive sharp waves, complex repetitive discharges, early recruitment, and small polyphasic motor unit potentials [3], but these findings are nonspecific and can be seen in other muscle diseases. Magnetic resonance imaging (MRI) is also a sensitive study for the evaluation of DM. It may show areas of inflammation presented as muscle edema and, in late stages, muscle atrophy [3]. Muscle or skin biopsy is the definitive method of diagnosis. Muscle biopsy should be taken from weak muscles with areas of inflammation and guided by MRI and clinical examination. Muscle biopsy findings consist of perifascicular atrophy, capillary abnormalities and loss, and perimysial abnormalities [3]. Unlike DM, biopsy of IMND usually shows much less inflammation in the muscle tissue and increased evidence of muscle cell death or necrosis.

AIDP is an autoimmune process that is characterized by progressive areflexic weakness and sensory changes. The most common AIDP is Guillain-Barré syndrome (GBS), which is also seen in one in 100,000 people [5]. AIDP and its most common variable GBS are thought to be triggered by an immune response to a preceding infection that cross-reacts with peripheral nerve components because of molecular mimicry.

While it is difficult to prove a causal relationship, EBV may have triggered AIDP and/or myositis in this patient. It also could be that these entities happened simultaneously. EBV has been associated with AIDP in 5% to 28% of cases [6]. EMG is the most specific and sensitive tests for the diagnosis of AIDP [7]. Plasmapheresis and IVIG are the two main immunotherapy treatments for AIDP. Lumbar puncture helps exclude other diagnoses and confirms AIDP. It was not performed in our patient because of apixaban use to avoid further delay in treatment given severe presentation of weakness and dysphagia requiring prompt treatment. As mentioned previously, EMG study was found to be consistent with AIDP diagnosis in our patient.

DM management may include initially glucocorticoids (1 mg/kg/day up to a maximum dose of 80 mg/day up with gradual taper in four to six weeks and steroid-sparing agent) [3]. Pulse glucocorticoids of 1 gm/day for three days can be used for severely ill patients. Tapering glucocorticoids is based on clinical response and improvement in muscle enzymes level. Steroid-sparing agents are generally initiated with glucocorticoids to help taper down glucocorticoids and prevent relapse. These agents include azathioprine, mycophenolate mofetil, and methotrexate. There are no large randomized trials to compare efficacy at this time. Rituximab was also used [8] but was not considered in this patient given reports of the possibility of worsening AIDP after rituximab use [9]. IVIGs also have been used in life-threatening conditions as it has a more rapid onset of action [10]. Given the presence of AIDP, IVIG was the best and safest treatment option for our patient.

## Conclusions

The presentation of IM, particularly DM with rhabdomyolysis, necrotizing features, and AIDP, is extremely rare. To our knowledge, there are no other case reports in the literature describing a similar presentation. Diagnoses are suspected based on muscle weakness and areflexia. A multidisciplinary team approach is essential. Skin and/or muscle biopsy, EMG, and lumbar puncture confirm the diagnosis. IVIG is considered an effective and safe treatment option for both diseases.
